# The Role of Oxytocin in Polycystic Ovary Syndrome: A Systematic Review

**DOI:** 10.3390/cimb46060313

**Published:** 2024-05-25

**Authors:** Nicoletta Cera, Joana Pinto, Duarte Pignatelli

**Affiliations:** 1 Faculty of Psychology and Education Sciences, University of Porto, 4099-002 Porto, Portugal; up201707095@up.pt; 2 Research Unit in Medical Imaging and Radiotherapy, Cross I&D Lisbon Research Centre, Escola Superior de Saúde da Cruz Vermelha Portuguesa, 1300-125 Lisbon, Portugal; 3 Faculty of Medicine, University of Porto, 4099-002 Porto, Portugal; duarte@med.up.pt; 4 Department of Endocrinology, Centro Hospitalar Universitário de São João, 4200-319 Porto, Portugal

**Keywords:** oxytocin, PCOS, systematic review, fertility

## Abstract

Polycystic Ovary Syndrome (PCOS) is the most common endocrine disorder that affects women of reproductive age, representing the primary cause of anovulatory infertility. The nonapeptide oxytocin (OT) plays an important role in cognitive, emotional, and reproductive functions in human beings. Oxytocin receptors are expressed in several body parts, including the ovaries. Despite this, the possible role played by oxytocin in symptoms of PCOS is not clear. The present systematic review aimed at understanding the presence of possible oxytocin level alterations in PCOS, the connection between alterations of OT levels and the symptoms of PCOS, and the effect of oxytocin administration in PCOS. After a systematic search in the principal databases, eight studies, five human and three animal, were included. Four human studies and one animal study highlighted the role played by oxytocin in fertility issues related to PCOS. Three human and two animal studies investigated the role of body weight and OT levels. Studies that analyzed oxytocin basal levels in women agreed that PCOS is associated with a reduction in the serum level of oxytocin. Two human studies and one animal study agreed about lower levels of oxytocin, confirming a possible implication of the dysfunction of OT in the pathogenesis of PCOS.

## 1. Introduction

Polycystic Ovary Syndrome (PCOS) is the most common endocrine disorder that affects women of reproductive age [1]. Depending on the diagnostic criteria used, its worldwide prevalence ranges from 4% up to 20% [2]. PCOS is characterized by polycystic ovary morphology, androgen excess, and ovulatory dysfunction [3]. Because of its typical metabolic, reproductive, and psychological features, PCOS is a relevant public health concern [4]. In particular, if taken into consideration, about 75% of PCOS cases are estimated to be undiagnosed [5]. PCOS is associated with high levels of androgens, including dehydroepiandrosterone and androstenedione, of adrenal origin, as well as androstenedione and testosterone, of ovarian origin [6]. PCOS is also usually characterized by increased luteinizing hormone (LH) levels, elevated LH/FSH ratios [6,7,8,9], and low to normal follicle-stimulating hormone (FSH) levels [7,10]. However, other studies suggest there are no significant differences in LH/FSH ratios [11] between PCOS and control groups.

Furthermore, PCOS is the primary cause of anovulatory infertility [12], accounting for 80% of such cases [13,14]. Pregnant PCOS women, on the other hand, have a higher risk of developing gestational diabetes mellitus or suffering a first-trimester spontaneous abortion [15,16]. Interestingly, even though an elevated baseline LH/FSH ratio in PCOS was found to be related to poor ovulatory response, PCOS cases with elevated LH/FSH ratio were more likely to achieve a clinical pregnancy and live birth than women with normal LH/FSH [9]. Ovarian function and cycles are regulated by the hypothalamic–pituitary–ovarian (HPO) axis, particularly by GnRH and the Gonadotropins.

Since the discovery of its structure in the early 1950s [17], oxytocin (OT), a nine-amino acid neuropeptide [18], has captivated the attention of the scientific community for its role in several animal and human physiological functions. Immunohistochemical studies showed that OT is synthesized in the magnocellular cells of the paraventricular (PVN), supraoptic nuclei (SON), and parvocellular of PVN, along with a few neurons in the accessory nuclei of the human and animal hypothalamus, and is transported to the neurohypophysis where it is released in the blood circulation [19,20,21,22]. Moreover, OT is dendritically released by the hypothalamic neurons and then passively diffused to various target brain regions [23].

Tracing studies also showed long-range axonal projections of oxytocinergic neurons to several brain regions, such as the hippocampal C1 and C2 subregions and ventral subiculum, shell, and core of nucleus accumbens, island of Calleja, lateral globus pallidus lateral, lateral septal nucleus, medial amygdaloid nucleus, and prelimbic cortex [24,25]. The magnocellular PVN projections reach the posterior pituitary gland, where the OT is released in the bloodstream, acting as a hormone [26]. In this way, OT plays a central role as a neurotransmitter and peripherally as a hormone.

OT plays a crucial role in several behavioral and reproductive functions in human beings, such as breastfeeding [27], pregnancy, and parturition [28,29,30], but also in other processes, like bonding, decision-making [31], prosocial behavior [32,33], and physical activity [34]. It is also related to the pleasure associated with orgasm, both in males and females, being released in elevated quantities during this process [35,36].

OT receptors (OXTR) are expressed in several body parts, including the ovaries and prostate gland [37,38]. Specifically, OXTRs are found to be expressed in the granulosa cells and the small follicles in several mammal species, including humans [39], and OT also takes an unclear role in steroidogenesis [40].

Despite the growing interest in the role played by oxytocin in several human functions, its possible role in one or more psychological symptoms, such as depression, anxiety, and social cognitive impairments, or physical symptoms, such as metabolic dysfunctions or infertility, of PCOS [41] is still not clear or little studied. However, depression was found to be associated with OT levels [42], and animal evidence highlighted the improvement of depressive symptoms, such as immobility, after OT administration [43,44]. Moreover, increased OT serum levels were observed in patients with mood disorders [45], and were associated with depressive symptoms [42]. Intranasal oxytocin treatment improved sleep apnea and in general the sleep quality in patients affected by sleep disorders [46,47].

Obesity is one of the principal symptoms observed in PCOS women. Studies on mice with a deficiency of OT (OT^−/−^) showed that OT deficiency is responsible for increased body weight and abdominal fat [48,49]. Interestingly, OT^−/−^ and OXTR^−/−^ normophagic mice develop late-onset obesity. Moreover, low OT levels were observed in diet-induced obese mice, and high OT levels observed in synaptotagmin-4-deficient mice that are protected against obesity [50]. Exogenous OT administration is found to decrease weight and increase muscular tone [51]. OT and its stable analog, carbetocin, have a negative modulatory effect on adipogenesis, with the promotion of osteogenesis in in human multipotent adipose-derived stem cells [52].

In the present systematic review, we aimed to disentangle the role played by oxytocin in PCOS, taking into account all animal and human studies that have been published. In particular, we aimed to understand (i) the presence of possible alterations of basal plasmatic OT level in PCOS, (ii) in which manner a possible alteration of the OT plasmatic level can be related to the symptoms of PCOS, and (iii) the presence of a possible effect of OT administration in PCOS.

## 2. Materials and Methods

**Figure 1:**
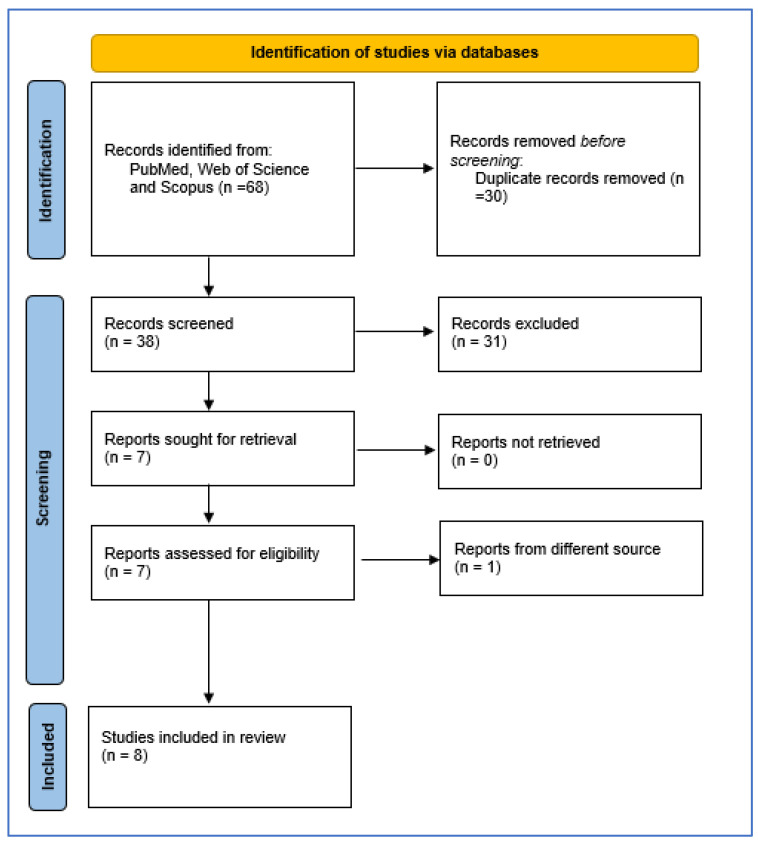
Flow chart of the selection process for PCOS and oxytocin. From: Page et al., 2021 [54] http://www.prisma-statement.org/ (accessed on 20 May 2024).

The present systematic review (PROSPERO reg. n. 531987) followed the procedure recommended by the Preferred Reporting Items for Systematic Reviews and Meta-Analyses (PRISMA) guidelines [53]. We performed a computer-based search in the principal databases, such as PubMed, Web of Science, and Scopus, combining terms related to polycystic ovary syndrome and oxytocin (Figure 1).

Moreover, our research question used the PICO strategy protocol. In particular, our research question was related to the role played by oxytocin (O—Outcome) in women or animal models with polycystic ovary syndrome (P—Population), determining the level of oxytocin in body fluids, such as blood and saliva (I—Intervention), in comparison with healthy women or animals (C—Comparison, Table 1, Supplementary Materials).

In the present systematic review, databases were selected to explore the published studies using the following keywords: “polycystic ovary syndrome” [MeSH Terms] OR (“polycystic” [All Fields] AND “ovary” [All Fields] AND “syndrome” [All Fields]) OR “polycystic ovary syndrome” [All Fields]) AND (“oxytocin” [MeSH Terms] OR “oxytocin” [All Fields] OR “oxytocin s [All Fields] OR “oxytocin [All Fields] OR “oxytocin [All Fields]”, with no time limit, and using the Boolean operators AND and OR. The inclusion and exclusion criteria were determined based on the topic, study design, and population (Table 2).

In the second stage, we removed the duplicates and manually screened both the titles and abstracts to evaluate if they fulfilled the inclusion and/or exclusion criteria. After that, we retrieved the full texts of the possibly pertinent studies to verify their eligibility. Two authors independently carried out the literature search, article screening, and methodologic evaluation. Both authors discussed the results and a consensus was reached. However, a third opinion was required when a consensus was not reached.

The included studies were subsequently screened to find further articles in the reference lists related to the topic of interest. Similarly, we screened all the excluded studies to identify additional relevant bibliographic sources. To estimate the quality of the selected studies, when possible, in the current systematic review, the NOS (Newcastle–Ottawa Scale), a quality assessment scale for case-control and cohort studies (https://www.ohri.ca/programs/clinical_epidemiology/nosgen.pdf (accessed on 1 March 2024), Tables S1 and S2, Supplementary Material), was used.

Then, information associated with the characteristics of the participants and inclusion and exclusion criteria were extracted from each included article, according to the previously mentioned guidelines. The flowchart in Figure 1 depicts the steps of the selection process.

## 3. Results

The flowchart depicted in Figure 1 shows the selection process of the studies. We included eight published studies in the systematic review, after reaching a consensus. Moreover, we calculated Cohen’s k with 87.17% (k = 0.742), indicating substantial agreement (https://idostatistics.com/cohen-kappa-free-calculator/ (accessed on 20 May 2024)) [55]. The characteristics of the studies are shown in Table 3.

Both human and animal studies were included. However, the selected studies did not show homogeneity in terms of both study design and population. The five human studies included 609 women and were published between 2010 and 2023. Among these, two were randomized or pseudo-randomized clinical trials, two were case-control studies, and another one was a population genetics study.

Moreover, the principal focus of the included studies was related to the relationship between fertility and nasal oxytocin administration. Similarly, Ochsenkühn et al. included patients with PCOS as a cause of infertility together with different infertile groups [60]. Conversely, the clinical trial performed by Masrour et al. [59] included only infertile patients with PCOS [56]. During the clinical trial, the patients underwent eight units of OT, but the authors did not observe any significant changes in terms of infertility. Ochsenkühn et al., who did not observe any improvement in the fertility of the PCOS group after OT treatment [60], obtained similar results. Despite the administration of OT, these two above-mentioned studies did not assess the level of blood or salivary oxytocin in the participants [60,61]. However, since the main topic of both RCTs was to assess the effect of eight IU of intranasal OT on infertility in PCOS, the studies found the treatment not relevant to improving fertility in PCOS patients affected by infertility. As underlined by Ochsenkühn et al., the failure to detect the effect of OT on the pregnancy rate could be the result of inadequacy in dose and or mode of administration [60]. The level of OT in PCOS before a treatment can be relevant, but none of the two RCTs collected such samples in their PCOS groups. However, Jahromi et al. compared the level of oxytocin and other hormones (Table 3) in infertile women both with or without PCOS [57]. These authors found that in PCOS, the mean level of OT was inferior to the non-PCOS group, with a mean value of 124.94 ng/L compared to 207.42 ng/L (*p* < 0.0001). Moreover, since the anti-mullerian hormone usually shows high levels in PCOS, it was negatively correlated with oxytocin, and the same occurred with insulin resistance. However, the authors did not observe a significant effect of BMI on oxytocin in both groups. According to the authors, the hormonal imbalances in the hypothalamic–pituitary–ovarian (HPO) axis, namely the high LH and low FSH in the PCOS group, could be connected to the lower oxytocin levels. These low levels can in turn be implicated in chronic anovulation. Notably, this study was the first that proposed a cut-off value of the oxytocin level in women with PCOS.

Similar results were observed in a case-control study [58] assessing the hypothalamic–pituitary–ovary axis dysfunction in a sample of 56 infertile PCOS women before ovarian stimulation with 2.5 mg of letrozole and before human chorionic gonadotropin (hCG) administration. The authors assessed the serum levels of OT, dopamine (DA), phoenixin-14 (PNX-14), and nesfatin-1 (NEF-1) in groups of pregnant and non-pregnant PCOS women. Moreover, FSH, LH, AMH, TSH, and prolactin were assessed. In the whole sample, they found a weak association of OT with BMI and a stronger one with FSH (*p* < 0.0002). However, in the pregnant group, higher baseline NES-1 and OT levels (+29.2% and +44%) were observed. Similarly, OT level increases were associated with positive pregnancy rates. After OS in pregnant women, the OT levels increased compared to non-pregnant women.

Finally, Amin and colleagues [56] assessed the presence of the polymorphisms of the gene responsible for the expression of the receptor of oxytocin (OXTR) in 212 Italian PCOS patients. OXTR is widely expressed in the human body, including in the brain and ovary tissue [64]. In their genetic population study, the authors tested the hypothesis whereby the OXTR variants are in linkage disequilibrium with PCOS in Italian families. They found that five variants, out of 22, were significantly (*p* < 0.05) linked to or were in linkage disequilibrium with PCOS. However, all of these variants were not previously related to clinical manifestations of PCOS. Nonetheless, three of them (rs60345038, rs35498753, and rs237900) were found to intersect with the repressed chromatin state in the ovaries, with a negative OXTR gene expression.

In the present systematic review, animal studies were included. The three animal studies included 50 female rats and were published in 2018 and 2022, respectively. These three studies were classified as RCTs and both administered OT to PCOS rat models. However, Sajadi et al. [61] also administered carbachol. Despite the use of OT, the main objective of the two studies was different. Sajadi and colleagues [61] studied the uterine contraction and tone in PCOS and non-PCOS rats after administration of OT or carbachol, while Yamamoto et al. [63] measured the effects of the administration of acute and chronic OT on metabolic disorders, as well as the changes in endogenous OT, in PCOS model rats.

Sajadi et al. [61] found that PCOS rats showed more irregular uterine contractions than controls, and that after being exposed to carbachol, their frequency and resting tone were significantly increased compared to controls. However, after the exposure to OT, there were no differences in frequency, resting tone, and amplitude of rhythmic contractions between both groups.

Yamamoto et al. [63] found that PCOS model rats showed lower serum OT levels than control rats. Nonetheless, the two groups did not differ in hypothalamic OT mRNA expression levels. The authors found that there were reductions in body weight gain and food intake only in PCOS model rats after acute intraperitoneal OT administration during the dark phase, whereas the chronic administration of OT decreased the food intake in both the PCOS model rats and control rats. Similar results have been observed by Isawa et al. [62]. They found no difference in the OT and OXTR mRNA expression in PCOS rats and PCOS rats treated with OT. The authors found that OT administration caused reductions in body weight, food intake, visceral fat weight, and adipocyte size in a DHT-induced rat model of PCOS. Among the included studies, one human study and two animal studies investigated the association between OT levels and BMI in humans or BW in animals (Table 4).

## 4. Discussion

Despite the relevant role played by the nonapeptide oxytocin in several functions, such as social cognition, metabolic regulation, and reproduction, only a few recent studies have investigated its role in PCOS. Given the novelty of the topic, the present systematic review took into consideration all the studies that investigated the role played by not only serum oxytocin but also randomized clinical trials with the administration of synthetic oxytocin. The present review also took into account animal studies, since most of the current knowledge about PCOS was obtained by studies on rat models. These played a crucial role in studying and gaining insights into human pathologies during the last two centuries. Despite some similarities between animals and humans, they also show distinct characteristics. Acknowledging important disparities regarding OT between animals and humans, the insights from rat studies are nevertheless important for the comprehension of the role played by OT in PCOS.

However, despite the lack of studies about the role played in PCOS and its related symptoms or comorbidities, such as metabolic syndrome, obesity, cardiovascular risk, and mood disorders [41], several studies have been conducted disentangling the possible role of serum OT in these pathological conditions. One of the comorbidities is represented by metabolic syndrome. Among women affected by metabolic syndrome, those with prediabetes and type two diabetes showed significantly lower serum OT levels than those affected by metabolic syndrome without diabetes [65]. In obese women, lower levels of plasmatic OT than in non-obese women were observed [66]. Metabolic alterations were also studied in PCOS rats [63]. Rats to which OT was administered showed a significant decrease in weight and food intake [63]. However, this study did not quantify the lipolysis in the adipose tissue of the rats. Previous findings indicated that OT administration significantly reduced the area of adipocytes, the serum triglyceride, aspartate aminotransferase level alanine aminotransferase, and alkaline phosphatase in ovariectomized rats [67]. Moreover, according to the results reported by Yamamoto and colleagues [63], an OT-mediated mechanism was involved in obesity and food intake in the PCOS rats. However, in PCOS the authors found a decrease in serum OT levels and no difference in the hypothalamic mRNA expression of OT and OXTR after the acute systemic administration of OT. In peri- and post-menopausal rats, the administration of OT reduced the body weight and adipocyte size, without affecting the serum levels of hepatic enzymes [68]. The menopausal period is considered a risk factor for visceral adiposity and metabolic disorders. Several studies found reduced serum OT levels in pre- and postmenopausal women [69,70,71,72].

Several findings showed that oxytocin is useful for treating obesity and preventing metabolic disorders [73,74,75,76,77,78]. Among the variants observed in PCOS in the population study of Amin et al. [56], rs60345038 was also found to be relevant for type 2 diabetes [79], indicating a possible predisposition for diabetes in PCOS patients. OT values were found to be significantly lower in obese post-menopausal women compared to obese pre-menopausal women. Interestingly, significantly lower OT serum levels were observed between obese and normal-weight postmenopausal women [69]. Significant lower OT levels were also found in women with surgical menopause as compared to those with normal menopause [70]. During menopause and in T2D patients, the cardiovascular risk increases. OT and OXTR were found in the atria and ventricles of rat hearts and in the large blood vessels, indicating a possible autocrine and paracrine role played by OT, regulating the vascular tone and the atrial and ventricular load [80,81]. In ovariectomized rats with deprivation of ovarian hormones, a reduction of the OXTR mRNA levels in PVN subnuclei was observed causing autonomic dysregulation [82].

Most of the studies underestimated the relevance of the levels of OT in women or female rats with PCOS by not reporting possible OT basal level differences. Notably, only three studies found that in PCOS, the levels of OT were lower than in healthy controls. Indeed, two human studies and one animal study agreed about lower levels of PCOS, confirming a possible implication of OT in the pathogenesis of the syndrome, which could negatively affect the effect of OT administrations. Despite the complexity of the symptoms of PCOS, anovulatory infertility represents one of the most relevant issues. In PCOS, the normal follicular development is compromised and the ovaries contain an excess of small antral follicles. Estrogen is an extracellular signal that can regulate the expression of OXTR. The estrogen receptor (E2) is localized in the nucleus, cytoplasm, and mitochondria. The two E2 subtypes (E2 α and E2β) affect both OT and OXTR expression: E2 α induces OXTR expression, whereas the activated E2β induces OT transcription in rat brains [83,84]. 

E2 is secreted by the granulosa cells of developing antral follicles, upon which FSH controls the maturation and the selection of the dominant pre-ovulatory follicle and triggers the LH required for ovulation [85,86,87]. The effect of E2 is mediated by estradiol receptors α (E2 α) and β (E2 β), which showed a different expression in the granulosa cells in developing follicles.

A few studies in humans and primates have found that E2 β is the principal mediator of E2 in granulosa cells. Since E2 β is detected in both pre-antral and mature follicles. Thus, E2 is considered as a marker of follicular quality. In PCOS patients, the selection of predominant pre-ovulatory follicle is arrested, and the observed low levels of FSH, in PCOS, do not allow the stimulation of follicle maturation. Follicular fluid in PCOS women showed low levels of E2 [78,79]. However, this mechanism is still unknown [88].

A significant increase of OT and E2 has been found in the myometrium during labor in healthy women, indicating a possible paracrine OT stimulation by E2 relevant to the uterus [89]. According to Pirog and colleagues, OT can be considered a predictor of pregnancy before ovarian stimulation therapy [58]. However, a recent meta-analysis assessed the OT concentrations during the stages of the menstrual cycle in normal cycling women, finding an increase in serum OT during the follicular phase. Interestingly, the change in oxytocin concentrations from the follicular phase to ovulation was larger than the change from the follicular phase to the luteal one [90]. In the follicular fluid of infertile women without PCOS, Tachibana et al. [91] found that the OT level was extremely low, and not related to the OT serum level. OT and E2 serum levels increased during ovulatory and luteal phases [91]. However, Franik et al. [92] found that values of E2/T and E2/A indexes, relevant to homeostasis model assessment of insulin resistance (HOMA-IR), were significantly lower in the PCOS than non-PCOS subjects, but did not differ significantly between the obese and normal weight groups.

The focus of the studies that we included was only related to the infertility issues related to PCOS. In different animal models, oxytocin seems to have a role in fertility by promoting the release of PGF2α from endometrial cells. Moreover, OT is involved in the process of luteolysis [93,94]. A similar mechanism is also present in humans, but a positive role of administration of OT in folliculogenesis and increasing pregnancy rates in both humans and animals was reported in a few studies [95,96]. Ochsenkühn and colleagues [60] did not observe any increase in the pregnancy rate in couples affected by PCOS and infertility after eight IU intranasal OT administrations. The role of OT in uterine contractions has been assessed in a recent study [61] which compared PCOS and non-PCOS female rats’ uteri. After administration of the oxytocin, no significant differences were observed in the amplitude, tone, and frequency in the rhythmic uterine contractions of PCOS rats. However, the increase in the dose of OT stimulated higher levels of tone, with a decrease in the contraction frequency in PCOS rats’ uterine tissues. It is well-studied that in the myometrium, the number of oxytocin receptors increases during pregnancy [97], allowing the uterus to become more sensitive to oxytocin and thus affecting the pattern of contractions during pregnancy and labor. Despite this, Leonhardt et al. [98] did not find uterine morphological differences using magnetic resonance imaging, and less uterine peristaltic movement was found in PCOS assessed with transvaginal ultrasonography.

Despite the increase in the number of receptors, their genetic expression can be different in PCOS. Amin et al. [56] reported five novel genetic variants for the receptor of OT (OXTR) associated with the risk of developing PCOS in multigenerational Italian families. These variants of the OXTR gene were found to be related to the principal symptoms of PCOS, such as anovulation or oligovulation, hyperandrogenism, polycystic ovaries, and the increased risk for metabolic alterations [79].

Some of the variants found by Amin are considered relevant for vulnerability to different disorders. The variant OXTR rs237902 found by Amin et al. [56] has been associated with schizophrenia vulnerability. Specifically, a significant association between rs237902 and negative symptoms, such as blunted affect, alogia, avolition asociality, and anhedonia, in schizophrenic patients and an overrepresentation in male aggressive children were observed [99,100,101].

The same variant (rs237902) was found to be related to autism spectrum disorder [102]. According to Dinsdale and Crespi [103], the relevance of oxytocin and possible alterations in the OT and OTXR system in PCOS is still not well understood. According to their review, PCOS and autism spectrum disorder (ASD) share several behavioral features that could induce speculation about a possible common role of OT in the two disorders [103]. Similarly, a recent meta-analysis [104] assessed the studies that reported the odds of PCOS women having a child with autism spectrum disorder and the risk of ASD in women with PCOS. The authors included ten studies finding that, according to the available evidence, PCOS women have increased odds of having a child with ASD. Regarding the evidence on the prevalence of ASD in PCOS women, results suggest that women with PCOS are more likely to be diagnosed with ASD. Particularly, Hergüner et al [105], after administration of the Autism-Spectrum Quotient (AQ) to a sample of PCOS women, found that patients showed higher total AQ and communication scores than age- and BMI-matched healthy women.

AQ is a 50-item self-report questionnaire that assesses social skills, communication skills, imagination abilities, attention switching, and attention to detail. AQ shows good psychometric properties.

## 5. Conclusions

PCOS is a multifaceted syndrome involving several symptoms affecting the patients’ quality of life at different levels. The present review described the studies that analyzed the levels, or the effects of administering, OT in PCOS.

The studies reported in the present systematic review took into account only a part of the possible roles played by OT in PCOS. Most of the studies highlighted the role played by OT in fertility issues related to PCOS, and only one study found an increased pregnancy rate concomitant with high OT levels. However, studies that analyzed the basal levels of OT in PCOS women agreed that is accompanied by a reduction in the serum level of oxytocin. One could speculate that OT acts in synergy with FSH to promote follicular development towards ovulation and low levels of OT in PCOS which, together with low levels of FSH, may contribute to the anovulation that is typical of PCOS. Another possible interplay occurs between OT levels and the ovulatory LH surge, suggesting a synergy in ovulation. This also might explain why PCOS cases that have low levels of OT do not have normal rates of ovulation.

However, less still is known about possible molecular mechanisms that may also be able to affect the central tone, resulting in cognitive and behavioral alterations in normal and PCOS women. The possibility of altered OXTRs in PCOS resulting from SNP variants supports this view.

Based on previous, non-PCOS evidence, a possible OT autocrine/paracrine imbalance in the granulosa cells may be hypothesized, influenced by anovulation that is observed in the PCOS phenotype A, in which the polycystic ovary morphology is present.

Further, the partial deprivation of ovarian hormones, as shown by studies in ovariectomized rats, can affect the OTXR mRNA hypothalamic levels, resulting in behavioral alterations and obesity.

It was noticeable that different dosage regimens of OT administration or OT administration patterns were not sufficiently studied. Furthermore, despite the novelty and relevance of the topic, none of these studies analyzed the effect of OT administration on prosocial behavior or in couples’ relationships and sexual satisfaction in PCOS.

## 6. Future Directions

Several outstanding issues need to be clarified by further studies that could disentangle the role played by OT in follicular development and ovulation/anovulation. Moreover, if the administration of OT, and in which dose and pattern of administration, can result in the improvement of ovulation in PCOS women should be investigated. Further studies are needed to clarify if OT basal serum levels are associated with metabolic disorders in PCOS. Moreover, none of the studies that were included in the present systematic review assessed the relationship between OT and social behavior and psychiatric comorbidities, such as mood and anxiety disorders, in PCOS and non-PCOS women.

## Figures and Tables

**Table 1:** The search strategy used in the present systematic review.

**Polycystic Ovary Syndrome**
1. Polycystic ovary syndrome [MeSHTerms]
2. Polycystic [All Fields]
3. Ovary [All Fields]
4. Syndrome [All Fields]
5. Polycystic ovary syndrome [All Fields]
OR/1-2; 4-1
AND/3-4; 1-10
**Oxytocin**
6. Oxytocin [MeSH Terms]
7. Oxytocin [All Fields]
8. Oxytocin s [All Fields]
9. Oxytocin [All Fields]
10. Oxytocins [All Fields]
OR/6-10

**Table 2:** Inclusion and exclusion criteria.

Inclusion Criteria	Exclusion Criteria
✓ Experimental studies ✓ Randomized clinical trials ✓ Clinical pilot studies ✓ Case-control ✓ Population genetics studies ✓ Animal studies	✓ Other endocrine diseases ✓ Male studies ✓ Reviews (scoping, narrative, and systematic) ✓ Meta-analyses

**Table 3:** Demographics, design, assessment, and principal results as shown in the included studies.

Source	Country	Subjects	Age	Design	Assessment	Treatment	Results
Amin et al., 2023 [56]	Italy	212 women	-	Population genetics study	Single nucleotide polymorphisms (SNPs) within OXTR.	-	Out of 22 OXTR-risk variants tested, three independent variants were significantly linked to/in LD with PCOS. Three intronic variants were linked to PCOS. One intronic variant and a synonymous variant were both linked and associated with PCOS. All variants are novel and have not been previously associated with PCOS or any PCOS-related phenotype. Three of the variants were found to confer risk for PCOS, intersected with a repressed chromatin state in the ovaries.
Jahromi et al., 2018 [57]	Iran	161 women (PCOS = 80; Non-PCOS = 81)	20–35 years	Case-control	OT, AMH, BMI, LH, T, FSH, TSH, prolactin, and DHEAS. Fasting blood sugar, fasting insulin, blood sugar 2 h after 75 g glucose, insulin 2 h after 75 g glucose, and HOMA-IR.	-	The mean OT level was lower in the case group. The mean BMI, AMH, HOMA-IR, fasting insulin, and insulin 2 h after 75 g glucose were higher in the PCOS group. OT was negatively correlated to AMH when evaluated for all participants or only among controls. OT was also negatively correlated to HOMA-IR among all participants. There was not a significant relationship between OT and BMI. The calculated cutoff value for OT was 125 ng/L and for AMH was 3.6 ng/mL in the PCOS group.
Piróg et al., 2023 [58]	Poland	56 infertile women with PCOS, 18 pregnant	31.89 ± 4.59 years	Case-control	Assessment before ovarian stimulation (OS) and before hCG administration. Assessments of PNX-14, NES-1, DA, and OT serum levels were performed. Other tests: LH, FSH, estradiol, PRL, AMH, and BMI.		In the whole cohort of patients, OT levels were weakly associated with BMI (r = 0.26, *p* = 0.048) and FSH (r = 0.47, *p* = 0.0002). Pregnant group: positive correlations between baseline OT and PRL (r = 0.47; *p* = 0.04), as well as OT and NES-1 (r = 0.55; *p* = 0.02). OT level increases were associated with positive pregnancy rates. In the post-OS, in pregnant PCOS, OT was 2.7 times lower than for non-pregnant women.
Masrour et al., 2018 [59]	Iran	150 women	19–39 (29 ± 4.48) years	Clinical trial	OT, HCG, FSH, prolactin, follicle number, and progesterone.	The three groups at random received: 100 mg clomiphene-citrate + 8 units of OT; 100 mg clomiphene-citrate + 10,000 units of HCG; 100 mg clomiphene citrate + 8 units of OT + 10,000 units of HCG.	There was no major difference among the groups regarding the ovulation rate or the number of follicles, nor were there any significant side effects observed in any groups.
Ochsenkühn et al., 2010 [60]	Germany	86 women	18–42 (34.2 ± 4.3) years	Randomized, double-blind, placebo-controlled clinical pilot study	Follicle number, double endometrial width, estradiol, LH, and progesterone. To assess male fertility: Semen parameters (native sperm concentration, progressive motility, normal sperm morphology, semen volume, and total progressive motile sperm count).	132 homologous IUI cycles with nasal application of placebo or eight IU OT following IUI.	In 132 IUI cycles of 86 women, 17 pregnancies were achieved, accounting for a pregnancy rate of 12.9% per IUI cycle. The pregnancy rates were 13.4% per IUI cycle in the placebo group, and 12.3% per IUI cycle in the OT group. As such, the difference was not statistically significant. No relevant side effects were observed in both groups.
Sajadi et al., 2018 [61]	Iran	14 female rats (PCOS = 7; Control = 7)	75–95 days	Randomized clinical trial	CCh; OT.	Rats in the experimental group were subcutaneously injected with 5 m/g of free testosterone on gestational day 20; controls received solvent. The contractions of isolated uterus in offspring of both groups were recorded by the power lab system, after exposure to CCh and OT.	Uterine contractions were more irregular in PCOS rats than controls, after exposure to both contractile agonists.
Isawa et al., 2019 [62]	Japan	Female rats: Seven PCOS–OT rats: DHT-treated rats (PCOS) receiving 380 μg/day OT. Six PCOS–saline rats: without OT treatment, treated with saline solution. Seven saline rats: non-DHT-treated rats with only saline treatment.	26 days	Randomized clinical trial	Serum levels of AST, ALT, and LDH. Histological analysis of ovaries and adipocytes. Hypothalamic mRNA levels of NPY, POMC, OT, and OXTR.	At 8 weeks, seven PCOS rats were implanted with OT (380μg/day)-filled minipumps which supplied 12 μL/day for 14 days. Six PCOS rats and seven control rats were implanted with saline-filled minipumps.	Body weight changes were significant between PCOS–OT and PCOS–saline rats, with PCOS rats being lighter. The mean visceral fat weight of the PCOS–OT rats did not differ from that of the saline-control rats. No difference in the number of cystic follicles was seen between the PCOS–OT and PCOS–saline rats. No difference in hypothalamic mRNA expression of NPY, POMC, OT, and OXTR among the three groups. No difference in AST and ALT among the three groups. LDH levels were higher in PCOS–saline rats than in the other two groups.
Yamamoto et al., 2022 [63]	Japan	16 female rats (PCOS_Chronic_ = 8; Control_Cronic_ = 8; PCOS_Acute_ = 8; Control_Acute_ =8)	28 days	Randomized clinical trial	OT, serum level. Hypothalamic mRNA levels of NPY, POMC, OT, OXTR, prepro-orexin, and agouti-related protein. Visceral fat mRNA expression of OT and OXTR	At 10 weeks after the surgical day, all rats were injected with saline for seven consecutive days, then injected with OT (1200 µg/kg, 0.4 to 0.5 mL injection volume) for the following seven consecutive days.	The serum OT level was lower in PCOS model rats than in control rats, whereas the hypothalamic OT mRNA expression level did not differ between them. Acute intraperitoneal administration of OT during the dark phase reduced the body weight gain and food intake in PCOS model rats. However, these effects were not observed in control rats. In contrast, chronic administration of OT decreased the food intake in both the PCOS model rats and control rats.

Abbreviations: OXTR, oxytocin receptor; LD, linkage disequilibrium; PCOS, Polycystic Ovary Syndrome; OT, oxytocin; AMH, anti-mullerian hormone; BMI, Body Mass Index; LH, luteinizing hormone; T, total testosterone; FSH, follicle-stimulating hormone; TSH, thyroid-stimulating hormone; DHEAS, dehydroepiandrosterone sulfate; HOMA-IR, insulin resistance index; HCG, chorionic gonadotropin; IUI, intrauterine insemination; CCh, carbachol. PNX-14, phoenixin-14; NES-1, nesfatin-1; DA, dopamine; PRL, prolactin. AST, aspartate aminotransferase; ALT, alanine aminotransferase; LDH, lactate dehydrogenase; NPY, neuropeptide Y; POMC, proopiomelanocortin.

**Table 4:** BMI and body weight associations with OT levels and OT administration, as reported in the included studies.

Source	Assessment of BMI/BW	Effect of OT Administration on BMI/BW	Association of BMI/BW and OT Serum Levels
Amin et al., 2023 [56]	No	-	Risk variants in the OXTR gene pose an increased risk for obesity (BMI > 30).
Jahromi et al., 2018 [57]	Yes	-	The mean oxytocin level was lower and the mean BMI was higher in PCOS. More PCOS women had a BMI > 25 than controls. However, the relationship between BMI and oxytocin was not significant.
Piróg et al., 2023 [58]	Yes	-	BMI levels were assessed both for pregnant and non-pregnant women. No between group differences in BMI. OT levels were weakly associated with BMI (r = 0.26, *p* = 0.048).
Masrour et al., 2018 [59]	No	-	-
Ochsenkühn et al., 2010 [60]	No	-	-
Sajadi et al., 2018 [61]	No	-	-
Isawa et al., 2019 [62]	Yes	The PCOS–OT group was significantly lighter than the PCOS-saline group. BW changes seen in the PCOS–OT group were significantly smaller than in the PCOS–saline group. BW was not quantified in the text.	-
Yamamoto et al., 2022 [63]	Yes	Exogenous administration of OT during the dark phase reduced BW gain only in PCOS model rats, and seven days of OT administration showed no significant differences between PCOS and control rats.	-

Abbreviations: BMI, body mass index; BW, body weight.

## Data Availability

All the data are shown in the full text.

## Supplementary Materials

The following supporting information can be downloaded at: https://www.mdpi.com/article/10.3390/cimb46060313/s1, Ref. [106] are cited in Supplementary Materials file. Table S1: Newcastle—Ottawa quality assessment scale case control studies; Table S2: Newcastle—Ottawa quality assessment scale cohort studies.

## Abbreviations

PCOS	Polycystic Ovary Syndrome
OT	oxytocin
PVN	paraventicular nucleus
SON	supraoptic nucleus
LH	luteinizing hormone
CA	cornu ammonis
PGF2α	Prostaglandin F2α
LD	linkage disequilibrium
AMH	anti-mullerian hormone
BMI	Body Mass Index
T	total testosterone
FSH	follicle-stimulating hormone
TSH	thyroid-stimulating hormone
DHEAS	dehydroepiandrosterone sulfate
HOMA-IR	insulin resistance index
HCG	chorionic gonadotropin
IUI	intrauterine insemination
CCh	carbachol
PNX-14	phoenixin-14
NES-1	nesfatin-1
DA	dopamine
PRL	prolactin
SNPs	single nucleotides polymorphism
P4	progesterone
E2	estradiol
AST	aspartate aminotransferase
ALT	alanine aminotransferase
LDH	lactate dehydrogenase
NPY	Neuropeptide Y
POMC	proopiomelanocortin
E2/T	estradiol/testosterone ratio
E2/A	estradiol/androstenedione ratio
