# Supplementary material for: The Role of Oxytocin in Polycystic Ovary Syndrome: A Systematic Review
Source: Curr Issues Mol Biol. 2024 May 25;46(6):5223–41. doi: 10.3390/cimb46060313 (PMC11201948; doi:10.3390/cimb46060313)
Supplement: Supplementary file 1 [file cimb-46-00313-s001.zip › cimb-2979470-supplementary.pdf]

Table S1. Newcastle - Ottawa quality assessment scale case control studies

Hits	Selection	Comparability	Exposure
Masrour et al., 2018	**	--	**
Jahromi et al., 2018	****	**	**
Ochsenkühn et al., 2010	****	**	**
Piróg et al., 2023	***	**	**

Note: A study can be awarded a maximum of four stars for the Selection and three stars Exposure categories. A maximum of two stars can be given for Comparability.

Table S2. Newcastle - Ottawa quality assessment scale cohort studies

Hits	Selection	Comparability	Outcome
Amin et al., 2023	****	--	**

Note: A study can be awarded a maximum of six stars for the Selection and three stars Exposure categories. A maximum of two stars can be given for Comparability.

PICO Worksheet and Search Strategy Protocol

1. Define your question using PICO by identifying: Patient/Problem, Intervention, Comparison group and outcome:
Patient/Problem: Patients with Polycystic Ovary syndrome (PCOS)/ PCOS Animal Models
Intervention: Hormonal level measurement/ pharmacological treatment
Comparison: comparison of the patients/animals with healthy controls
Outcome: Treatment effects and /or difference in oxytocin levels
Write out your question
2. Type of question/problem: The role of oxytocin in PCOS.
Circle one: x Therapy/Prevention/Diagnosis/Etiology/Prognosis
3. Type of studies/publications to include in the search:
Check all that apply:
□ x Meta-analysis □ x Systematic review
□ Clinical practice guidelines □ xRandomized controlled trial
□ xResearch studies or articles □ Case report or series
□ Research report or other grey literature
4. List main topics and alternate terms from your PICO question that can be used for your search: “experimental studies”; “women”; “PCOS”; “rats”; “Oxytocin”.
5. Write out your search strategy: "polycystic ovary syndrome"[MeSH Terms] OR ("polycystic"[All Fields] AND "ovary"[All Fields] AND "syndrome"[All Fields]) OR "polycystic ovary syndrome"[All Fields]) AND ("oxytocin"[MeSH Terms] OR "oxytocin"[All Fields] OR "oxytocin s [All Fields] OR "oxytocin [All Fields] OR "oxytocin [All Fields]”
Boolean search operators (AND) and (OR).
6. List any limits that may apply to your search:
Gender: Female;
Age: Adults;
Year(s) of publication: no limits; Language(s): English
7. List the databases you will search: PubMed, Scopus, and Web of Science.

This form is adapted from: Miller, S.A. [106] . PICO worksheet and search strategy. US National Center for Dental Hygiene Research
